# Construction of a Cuprotosis-Related Gene-Based Model to Improve the Prognostic Evaluation of Patients with Gastric Cancer

**DOI:** 10.1155/2022/8087622

**Published:** 2022-09-22

**Authors:** Chunyan Han, Kai Zhang, XinKai Mo

**Affiliations:** ^1^Departments of Radiotherapy, The Third Affiliated Hospital of Shandong First Medical University, Jinan, 250031 Shandong, China; ^2^Departments of Medicine-Oncology, The Third Affiliated Hospital of Shandong First Medical University, Jinan, 250031 Shandong, China; ^3^Gastroenterology Research Institute and Clinical Center, Shandong First Medical University (Shandong Academy of Medical Sciences), Jinan, 250031 Shandong, China; ^4^Department of Clinical Laboratory, Shandong Cancer Hospital and Institute, Shandong First Medical University and Shandong Academy of Medical Sciences, Jinan, 250031 Shandong, China

## Abstract

**Background:**

Gastric cancer (GC) is one of the most serious gastrointestinal malignancies with bad prognosis. The association between GC and cuprotosis-related genes has not been reported.

**Methods:**

The clinical and RNA expression of patients with GC were downloaded from TCGA database. The CIBERSORT package was used to quantify the abundance of specific cell types. Using the Cox regression analysis, we conducted a prognostic nomogram model based on cuprotosis-related differential genes in GC. We evaluated the prognostic power of this model using the Kaplan-Meier (K-M) survival curve analysis, decision curve analysis (DCA), and receiver operating characteristic (ROC) curve analysis.

**Results:**

The plasma cells, monocytes, and mast cells in GC tissue were significantly less than those in adjacent tissue (*p* < 0.05), while T cell CD4 memory activated macrophage M0, macrophage M1, and macrophages in GC tissue. The number of M2 was significantly more than that in the adjacent tissue (*p* < 0.05). Additionally, GC patients in the test group, the training group, and all the sample groups had shorter survival time with the increase of the risk factor (*p* < 0.05). The nomogram of GC based on cuprotosis prognosis-related genes was conducted. The AUC of the nomogram to predict 1-, 3-, and 5-year survival rate was 0.618, 0.618, and 0.625, respectively.

**Conclusion:**

A novel cuprotosis-related gene signature impacts on the prognosis of GC. Our research provides new insights and potential targets for studying the link between GC and cuprotosis point, thereby providing new insights into understanding the molecular mechanism of GC.

## 1. Introduction

Gastric cancer (GC) is the main cancer of the gastrointestinal tract. According to relevant statistics, GC has become the fifth most common cancer in the world and the third leading cause of cancer-related death and has become one of the major global health problems [1–3]. GC account for approximately 95% of GC. According to anatomical location, GC can be divided into cardia/proximal and noncardia/distal; according to tissue type, it can be divided into intestinal type and diffuse type [4]. The early symptoms of GC are not obvious, resulting in a low diagnostic detection rate. Once found, it is often in the middle and late stages, resulting in a poor prognosis [5]. Therefore, it is necessary to seek effective early diagnosis and accurate prognosis prediction of GC.

Copper is an indispensable trace element involved in various biological processes. Recent studies have shown that copper levels in serum and tumor tissue are significantly elevated in cancer patients compared to healthy individuals [6, 7]. The concept of cuprotosis, proposed in 2022, occurs through the direct binding of copper to fatty acylated components of the tricarboxylic acid (TCA) cycle, leading to fatty acylated protein aggregation and subsequent loss of iron-sulfur cluster proteins; these result in proteotoxic stress and ultimately cell death [8]. And so far, no one has studied the relationship between cuprotosis and GC.

The tumor microenvironment (TME) has been confirmed to be closely related to the occurrence, growth, and metastasis of GC [9], and the tumor microenvironment is conducive to promoting GC immune and antitumor therapy [10]. The relationship between TME and GC still needs further exploration.

However, a comprehensive analysis of associated prognosis, tumor immune microenvironment, and immunotherapy based on cuprotosis has not yet been performed. In our study, based on the TCGA database and CIBERSORT, we performed immune infiltration correlation analysis in GC, screened cuprotosis-related differentially expressed genes, and constructed a cuprotosis prognostic model and its risk factor analysis. Prognostic features can effectively predict the prognosis of GC patients. At the same time, relevant functional analysis, immune microenvironment and immune-related function analysis, immune escape, immunotherapy, and screening of potential drugs were carried out. Our research provides new insights and potential targets for studying the link between GC and cuprotosis point, thereby providing new insights into understanding the molecular mechanism of GC.

## 2. Method

### 2.1. Screening of STAD Transcriptome Expression Matrix

The TCGA (https://portal.gdc.cancer.gov/) website was used to download clinical and transcriptomic expression data related to GC, and R software was used to sort, summarize and summarize the clinical data, and further obtain its expression matrix. And we used the Perl script to screen out the expression matrix of mRNA and remove the expression data of noncoding RNA.

### 2.2. Analysis of Immune Infiltration of GC

Using the CIBERSORT package to deconvolute the STAD expression matrix data obtained above, the cellular composition of complex tissues can be estimated based on normalized gene expression data, which can quantify the abundance of specific cell types. And get the STAD-related infiltrating immune cell expression matrix, and use the immune sorting Perl script to sort immune cells, in the GEO (gene expression omnibus) database (https://www.ncbi.nlm.nih.gov/geo/) to download the gene chip data related to GC, and the screening conditions are (1) GC, (2) human, and (3) GC tissue and adjacent tissue. Background correction, normalization, and expression value calculations were performed on the microarray data using the limma R package in R, and the CIBERSORT package was used to estimate the cellular composition of GC and adjacent tissue. The composition of immune cells in each sample was further analyzed using the CIBERSORT package, and histograms were drawn. A heatmap of immune cell distribution was drawn using the pheatmap package. Then, the corrplot package was used to analyze the interaction between immune cell populations in GC and to plot the coexpression of immune cell infiltration in GC. Finally, the vioplot package was used to analyze the expression of each immune cell in GC tissue and adjacent tissue and further draw the violin diagram of immune cell expression.

### 2.3. Establishment of Cuproptosis-Related Gene Expression Matrix in GC

The STAD mRNA expression matrix obtained in the previous stage was combined with the expression matrix of the currently known 19 cuprotosis genes, and the correlation heatmap was drawn using the limma package, and the differential expression between the two groups was calculated according to and using the limma package. In mRNAs, set *p* value < 0.05, and the expression change range ≥ 1.50 times (|log2FC| ≥ 0.58) is the criterion for screening differential genes, where log2FC ≥ 0.58 means mRNA expression is upregulated and log2FC ≤ −0.58 means mRNA expression is downregulated. Finally, the differentially expressed mRNAs related to cuprotosis between the STAD group and the control group were obtained, that is, the STAD differentially expressed genes (DEGs). The heatmap package was used to draw heatmaps and cluster analysis of the filtered DEGs, and the *p* value in the differentiated data was converted to -log10, and the -log10 (*p* value) was grouped according to log2FC (upregulated DEG group, downregulated DEG group, and DEG group with no statistical significance) and imported the processed data into *R* to draw a volcano plot.

### 2.4. Construction of a Cuprotosis Prognostic Model for GC and Analysis of Risk Factors

The standardized STAD cuprotosis-related expression data was merged with the clinical data of GC, and R language packages such as survivor, caret, glmnet, survminer, and survivorROC were used to perform univariate and multivariate Cox prognostic survival analysis of GC differential genes, dig out the key cuprotosis genes closely related to the prognosis of GC, and draw its survival curve. In addition, 443 cases were randomly divided into a test group (Test) and a training group (Train), and the survival package was called to perform risk-survival analysis on the combined data in the early stage, and the risk-survival curve and ROC curve were drawn, the training group (Train), and the survival state graph of all samples. The survival package was called again to carry out clinical statistical analysis and risk prognosis analysis on the general clinical data of GC. The univariate and multivariate independent prognostic analysis was used to mine the risk factors related to GC, and the univariate and multivariate independent prognostic analysis forest diagram was drawn, C-index curve, nomogram, and further survival, survminer package for model validation of clinical grouping.

### 2.5. Principal Component Analysis and GO and KEGG Enrichment Analyses of Cuproptosis-Related Genes in GC

The scatterplot3d package was used to carry out principal component analysis of GC cuproptosis-related genes and GC-related risk genes, and the clusterProfilerGO.R package and Perl language in R language (https://www.r-project.org/) software were used to analyze GC. The cuprotosis-related differential genes of cancer were subjected to GO analysis, respectively. GO analysis is mainly used to describe the function of gene products, including Cellular Component (CC), Molecular Function (MF), and Biological Process (BP). The clusterProfilerKEGG.R package was used for KEGG pathway enrichment analysis, and the enrichment degree of core pathways was analyzed according to the enrichment factor value, and the potential biological function and signaling pathway mechanism of GC were explored.

### 2.6. Immune-Related Function Analysis of Cuprotosis-Related Risk Genes in GC, Immune Escape, and Immunotherapy

limma, GSVA, GSEABase, pheatmap, and reshape2 packages were used to analyze the immune-related functions of risk genes related to cuprotosis in GC, in order to achieve precise treatment. In addition, the limma and ggpubr packages were used to conduct immune escape and immunotherapy-related analysis on the previously constructed GC cuprotosis-related prognostic model, in order to evaluate the effectiveness of immunotherapy for GC cuprotosis-related risk genes.

### 2.7. Tumor Mutation Burden (TMB) Analysis of Cuprotosis-Related Risk Genes in GC

The expression files of cuprotosis-related risk genes were constructed, the TMB files were downloaded from the database, and the correlation between core genes and TMB was tested by using the function, and the correlation, coefficient, and *p* value were calculated. In addition, the survival and survminer packages were used to analyze the correlation between cuprotosis-related risk genes and GC tumor mutation burden, and the correlation, coefficient, and *p* value were calculated.

### 2.8. Relative Expression of Core Target Genes

Download the expression matrix data of GC in TCGA, including the transcriptome expression matrix file in STAD (stomach adenocarcinoma), and use the ggpubr package to analyze the relative expression of the core targets in the STAD expression data based on the core targets screened in the previous stage, and plot the relative expression box plots of core targets.

### 2.9. Single-Gene GSEA Enrichment Analysis

Using limma (http://org.Hs.eg.db), clusterProfiler, and enrichplot packages to carry out GO and KEGG enrichment analyses of core genes, respectively, download the GO/KEGG annotation files of whole transcriptome genes from GSEA official website, and analyze the cellular components (Cellular Component, CC), molecular function (Molecular Function, MF), and biological process (Biological Process, BP) and KEGG pathway enrichment analysis. The enrichment degree of core pathways was analyzed according to the enrichment factor values, and the potential biological functions and signaling pathway mechanisms of core genes in GC were explored.

### 2.10. Potential Drug Screening

According to the preliminary screening of GC cuprotosis-related risk genes, the CPG2016 drug database was used to screen the therapeutic effects of these genes, and the limma, ggpubr, pRRophetic, and ggplot2 packages were run to evaluate effective drugs for GC cuprotosis-related risk gene therapy.

### 2.11. Statistical Analysis

All the statistical analyses and drawings in this study used R (version 4.2.1) or GraphPad Prism (version 8.3.0). A *t*-test was used to analyze differences between continuous variable. Fisher's exact test or chi-square test was employed for comparisons of categorical variables. Log-rank test was used to estimate the differences among K-M survival curves. *p* < 0.05 was considered significant.

## 3. Results

### 3.1. Analysis of Immune Infiltration of GC

**Figure 1:**
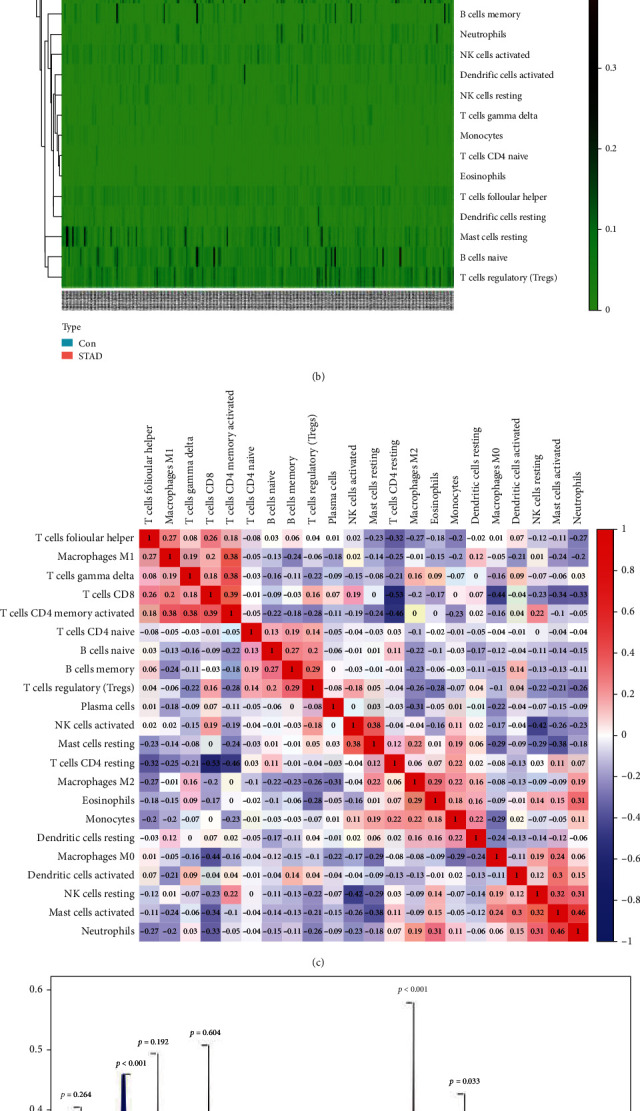
Analysis of immune cell infiltration. (a) Histogram of immune cell distribution in GC. (b) Heatmap of immune cell distribution in GC. (c) Heatmap of immune cell interaction in GC. (d) Violin plot of the relative content of immune cells in GC.

The obtained STAD expression matrix data was used for background correction, normalization, and expression value calculation of the chip data using the limma R package in R, and the CIBERSORT package was used to calculate the immune cell composition of GC and adjacent tissue. And further use the CIBERSORT package to analyze the composition of immune cells in each sample, and draw a histogram, as shown in Figure 1(a). The pheatmap package was used to draw a heatmap of immune cell distribution in Figure 1(b). Then, the corrplot package was used to analyze the interaction between immune cell populations in GC, and the coexpression map of immune cell infiltration in GC was drawn, as shown in Figure 1(c). Finally, use the vioplot package to analyze the expression of each immune cell in GC tissue and adjacent tissue, and further draw the violin diagram of immune cell expression, as shown in Figure 1(d). From Figure 1(d), the resting numbers of plasma cells, monocytes, and mast cells in GC tissue were significantly less than those in adjacent tissue (*p* < 0.05), while T cell CD4 memory activated macrophage M0, macrophage M1, and macrophages in GC tissue. The number of M2 was significantly more than that in the adjacent tissue (*p* < 0.05), while the number of other immune cells had no significant difference between the two groups (*p* > 0.05).

### 3.2. Differentially Expressed Genes Related to Cuprotosis in GC

**Figure 2:**
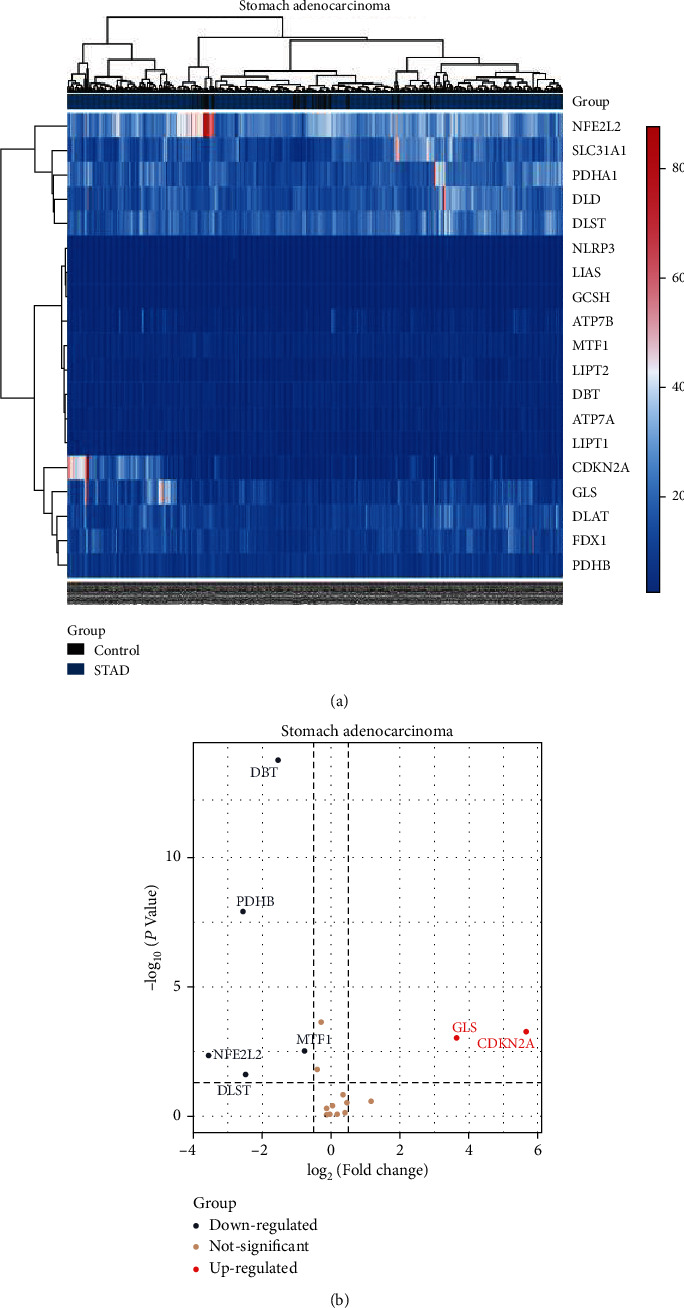
Differentially expressed genes of cuprotosis in GC. (a) Heatmap of STAD cuproptosis-related gene clustering. (b) Volcano map of STAD cuproptosis-related gene clustering.

Use the TCGA (https://portal.gdc.cancer.gov/) website to download the clinical and transcriptome expression data related to GC and obtain 407 transcriptome-related data sets and 443 clinically related data sets according to the preset screening conditions data set, use R software to organize and summarize clinical data, and further obtain its cuprotosis-related expression matrix. According to the *p* value < 0.05, the expression change range ≥ 1.5 times (|log2 FC| ≥ 0.58) was the criterion for screening differentially expressed genes, and 7 differentially expressed mRNAs were screened in the data set, including 2 upregulated mRNAs and 5 downregulated mRNAs, including DBT, PDHB, CDKN2A, GLS, MTF1, NFE2L2, DLST, and draw STAD cuproptosis-related gene expression matrix heatmap in Figure 2(a), wherein red represents upregulated gene expression and blue represents downregulated gene expression. The *p* value after the difference analysis was converted to -log10, and the -log10 (*p* value) was grouped according to log_2_ FC (upregulated DEG group, downregulated DEG group, and insignificant DEG group) and imported the processed data into R plot the volcano in Figure 2(b).

### 3.3. Construction of a Cuprotosis Prognostic Model for GC and Analysis of Risk Factors

**Figure 3:**
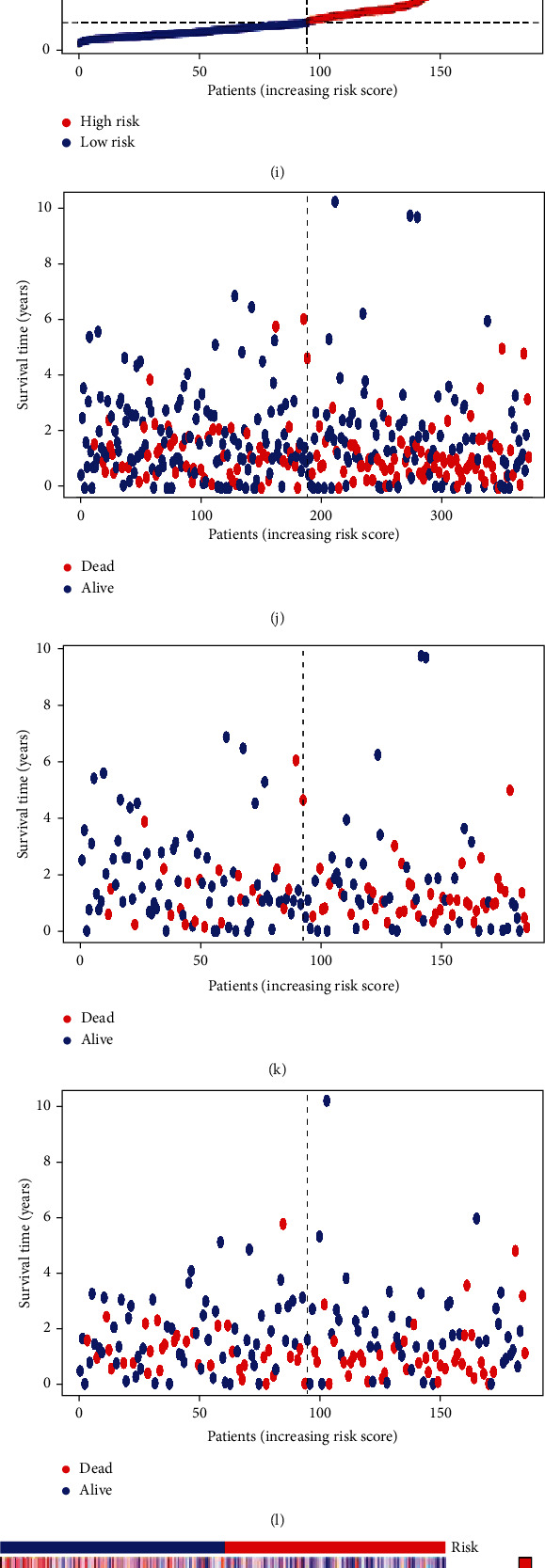
Construction of a GC prognostic model. (a) Survival prognosis curve of total sample group. (b) Survival prognosis curve of training group. (c) Survival prognosis curve of test group. (d) Progression-free survival prognosis curve. (e) Univariate regression independent prognosis analysis. (f) Multivariate regression independent prognosis analysis. (g) Risk score of total sample group. (h) Risk score of training group. (i) Risk score of test group. (j) Survival state diagram of total sample group. (k) Survival state diagram of training group. (l) Survival state diagram of testing group. (m) Heatmap of cuprotosis gene survival analysis in total sample group. (n) Heatmap of cuprotosis gene survival analysis in training group. (o) Heatmap of cuprotosis gene survival analysis in test group. (p) ROC curve of five-year survival of GC. (q) Risk of GC factor ROC curve. (r) GC C-index curve. (s) GC cuprotosis prognostic model nomogram.

443 clinical cases were randomly divided into a test group (Test) and a training group (Train), and the clinical statistical analysis of the general clinical data of the two groups found that there was no significant difference in age, gender, and tumor stage between the two groups (*p* > 0.05); the two groups of data are comparable in Table 1. The Cox survival prognostic model was constructed, respectively, and it was found that the three groups of GC patients in the test group (Test), the training group (Train), and all the sample groups had shorter survival time with the increase of the risk factor (*p* < 0.05), as shown in Figures 3(a)–3(c). The analysis of progression-free survival showed that the survival time of GC in the high-risk group was significantly shorter than that in the low-risk group (*p* < 0.05), as shown in Figure 3(d). And through univariate and multivariate regression independent prognostic analysis, it was found that tumor stage and risk factor were risk factors for GC, as shown in Figures 3(e) and 3(f). In addition, the survival status of the test group (Test), the training group (Train), and all samples was analyzed, and it was found that with the increase of the risk factor, the death rate of GC patients increased, as shown in Figures 3(g)–3(o). In addition, by constructing 1-year, 3-year, and 5-year ROC curves, it was found that the area under the three groups of curves, AUC, was greater than 0.6, as shown in Figure 3(p). In addition, by constructing the ROC curve of GC risk factors, it was found that the area under the curve AUC of the cuprotosis risk gene was the largest and was greater than 0.6, as shown in Figure 3(q); it indicated that the established survival prognosis model had better sensitivity. In addition, by constructing a C-index curve, it was found that both cuprotosis risk genes and tumor stage are important indicators for evaluating the prognosis of GC, as shown in Figure 3(r). Finally, by constructing a nomogram of the GC cuprotosis prognosis model, it was found that the 1-year survival rate was 0.827, the 3-year survival rate was 0.545, and the 5-year survival rate was 0.425 (*p* < 0.05), as shown in Figure 3(s).

### 3.4. Principal Component Analysis and GO and KEGG Enrichment Analyses of Cuproptosis-Related Genes in GC

**Figure 4:**
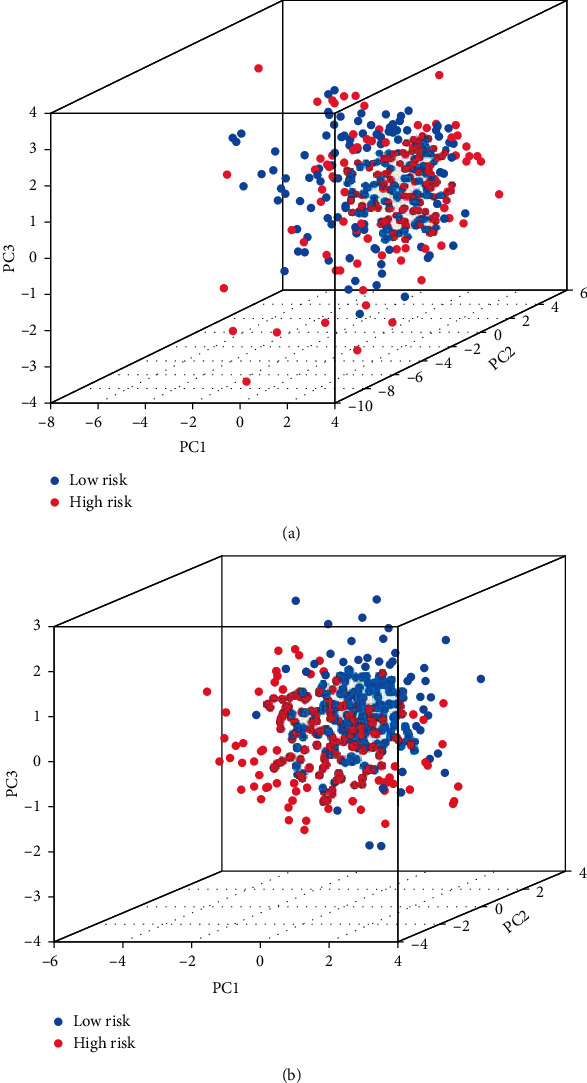
PCA distribution map. (a) PCA map of cuproptosis-related genes. (b) PCA map of GC-related risk genes.

**Figure 5:**
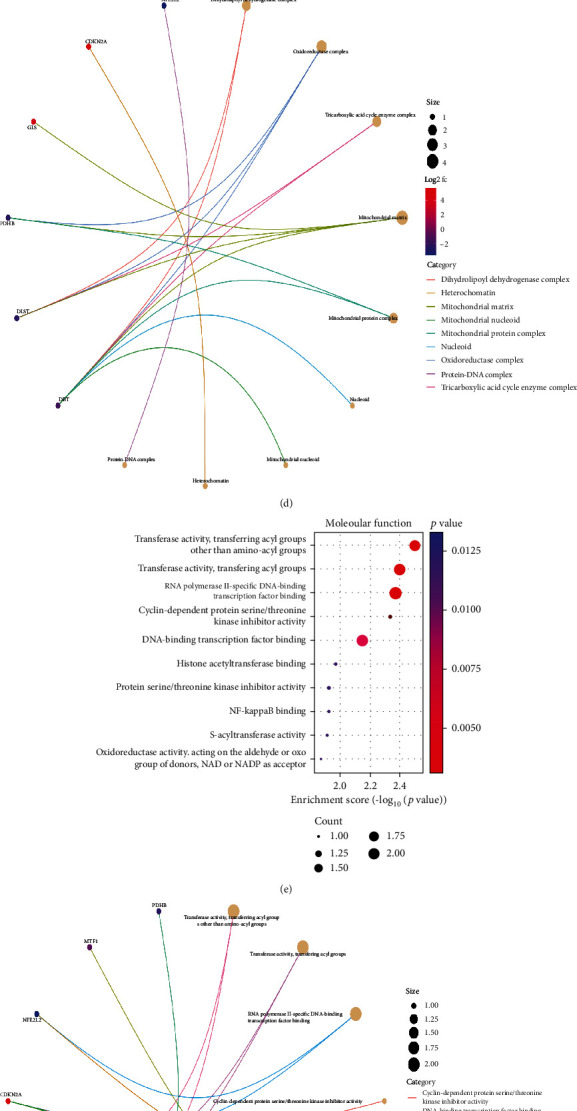
Enrichment analysis. (a) BP enrichment bubble map. (b) BP enrichment arc map. (c) CC enrichment bubble map. (d) CC enrichment arc map. (e) MF enrichment bubble map. (f) MF enrichment arc diagram. (g) KEGG enrichment bubble diagram. (h) KEGG enrichment arc diagram.

The scatterplot3d package was used to perform principal component analysis on cuproptosis-related genes and GC-related risk genes in GC. The PCA diagrams are shown in Figures 4(a) and 4(b). Using the Bioconductor package and clusterProfiler package in R language, GO and KEGG pathway enrichment analyses of 7 differentially expressed genes related to cuprotosis in GC were carried out. The results showed that GO analysis of seven potential target genes showed that their biological processes were mainly enriched in cellular amino acid catabolic process, tricarboxylic acid cycle, and acetyl-CoA metabolic process, the cellular components were mainly enriched in dihydrolipoyl dehydrogenase complex, oxidoreductase complex, and tricarboxylic acid cycle enzyme complex, and the molecular functions are mainly enriched in transferase activity, transferring acyl groups other than amino-acyl groups, transferase activity, transferring acyl groups, and RNA polymerase II-specific DNA-binding transcription factor binding in Figures 5(a)–5(f). KEGG pathway enrichment analysis found that it is mainly concentrated in citrate cycle (TCA cycle), central carbon metabolism in cancer, carbon metabolism, hepatocellular carcinoma, and arginine biosynthesis in Figures 5(g) and 5(h).

### 3.5. Immune-Related Function Analysis of Cuprotosis-Related Risk Genes in GC, Immune Escape, and Immunotherapy

**Figure 6:**
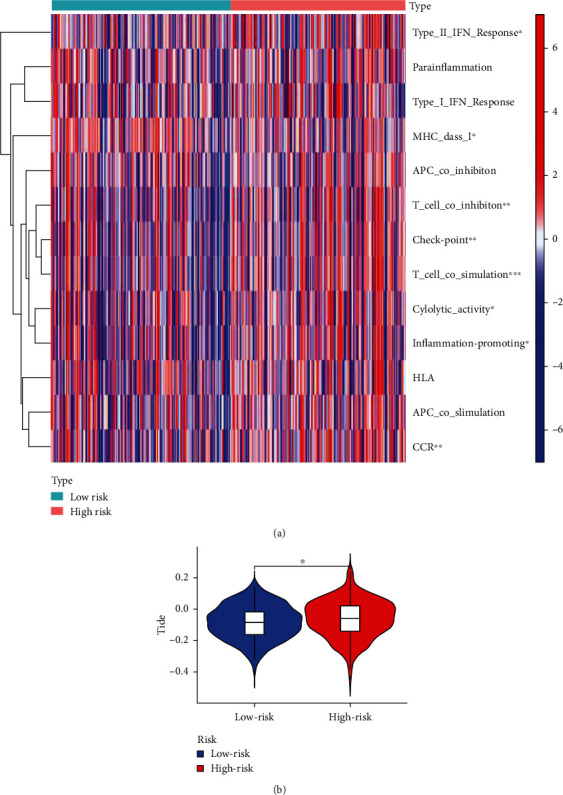
Analysis of immune function. (a) Heatmap analysis of immune-related functions. (b) Immune escape and immunotherapy of GC-related risk genes.

Using limma, GSVA, GSEABase, pheatmap, and reshape2 packages to analyze the immune-related functions of cuprotosis-related risk genes in GC, it was found that the immune functions of GC were mainly concentrated in APC coinhibition, APC costimulation, CCR, checkpoint, cytolytic activity, HLA, inflammation-promoting, MHC class I, parainflammation, T cell coinhibition, T cell costimulation, type I IFN reponse, type II IFN reponse, etc. The relevant heatmap is shown in Figure 6(a).

### 3.6. Tumor Mutation Burden (TMB) Analysis of Cuprotosis-Related Risk Genes in GC

**Figure 7:**
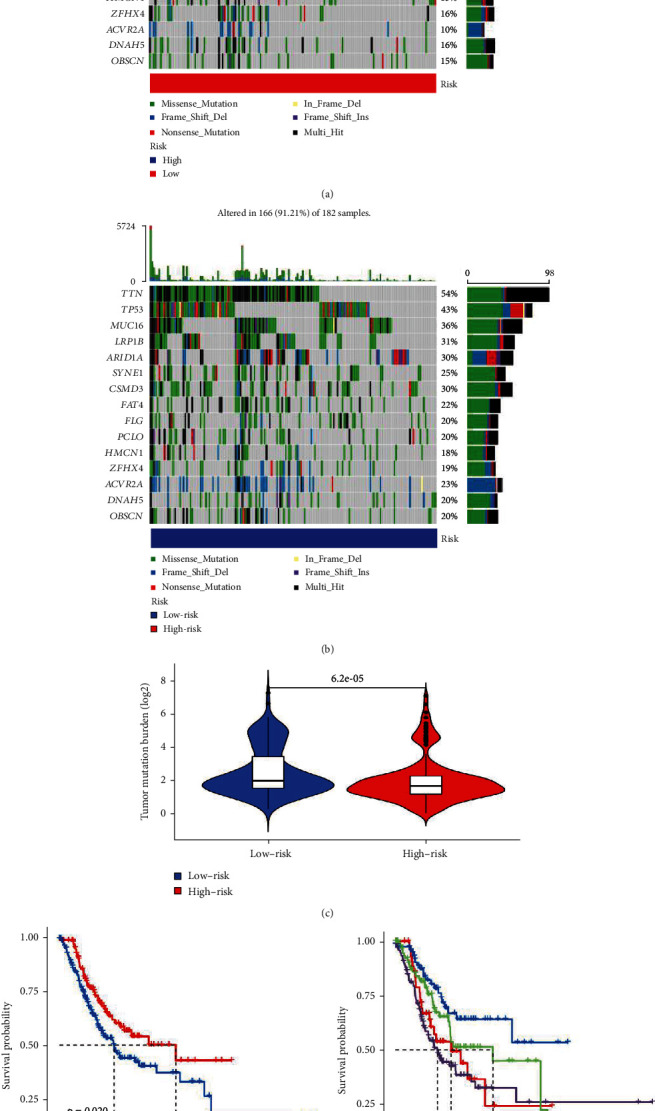
TMB analysis. (a) Waterfall diagram of high-risk group. (b) Waterfall diagram of low-risk group. (c) Difference analysis diagram of tumor mutation burden. (d) Survival analysis of tumor mutation burden. (e) Survival analysis of TMV+ risk factors.

Import the STAD expression data and TMB file into R, and use the function to calculate the correlation between GC cuprotosis-related risk genes and STAF tumor mutation load, and draw waterfall charts for high- and low-risk groups according to the correlation results, as shown in Figures 7(a) and 7(b). The possibility of tumor mutation burden in the low-risk group was greater than that in the high-risk group, and the difference analysis of tumor mutation burden showed that the tumor mutation burden in the low-risk group was significantly higher than that in the high-risk group (*p* < 0.05), as shown in Figure 7(c). In addition, survival analysis of the tumor mutation burden of risk genes related to cuprotosis in GC showed that the survival probability of the high tumor mutation burden group was significantly longer than that of the low tumor mutation burden group over time (*p* < 0.05) in Figure 7(d). In addition, combining the characteristics of tumor mutation burden and risk gene factors related to cuprotosis, it was found that the high tumor mutation burden combined with low-risk group had the highest probability of survival, while the low tumor mutation burden combined with high-risk group had the lowest probability of survival, as shown in Figure 7(d).

### 3.7. Relative Expression of Core Target Genes

**Figure 8:**
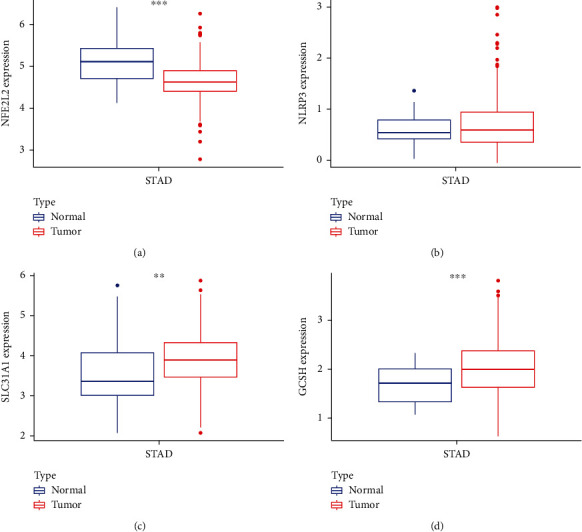
Relative expression of core target genes. (a) Relative expression of NFE2L2. (b) Relative expression of NLRP3. (c) Relative expression of SLC31A1. (d) Relative expression of GCSH.

The core cuprotosis risk genes NFE2L2, NLRP3, SLC31A1, and GCSH were obtained through differential expression analysis and survival prognosis analysis in the early stage. We further studied the relative expression of core cuprotosis risk genes NFE2L2, NLRP3, SLC31A1, and GCSH in GC STAD. Download the relevant expression data of STAD from TCGA, and analyze the relative expression levels of NFE2L2, NLRP3, SLC31A1, and GCSH genes through the ggpubr package, and draw a box expression map, as shown in Figures 8(a)–8(d). The results showed that NFE2L2 was lowly expressed in STAD tumor tissue (*p* < 0.001), while SLC31A1 and GCSH genes were highly expressed in STAD tumor tissue (*p* < 0.01), and there was no significant difference in the expression of NLRP3 between the two groups (*p* > 0.05).

### 3.8. Single-Gene GSEA Enrichment Analysis

**Figure 9:**
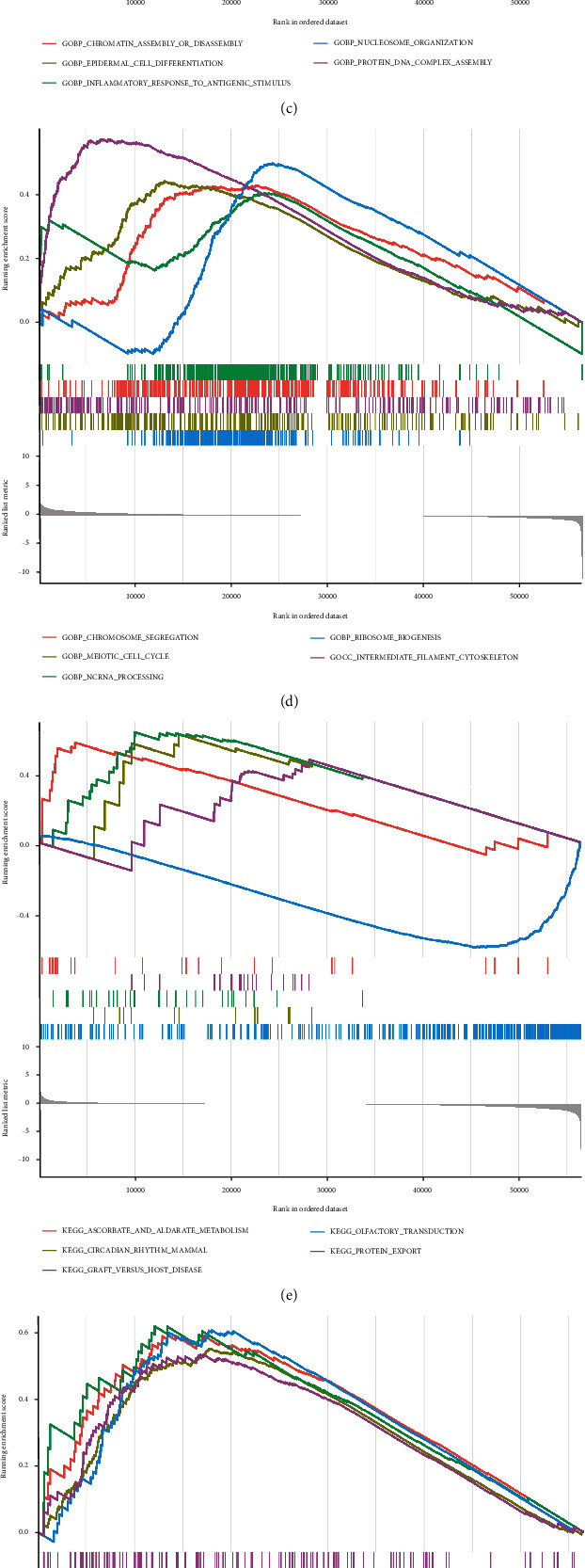
Single-gene GSEA enrichment analysis. (a) GO enrichment analysis in NFE2L2 gene STAD. (b) GO enrichment analysis in NLRP3 gene STAD. (c) GO enrichment analysis in SLC31A1 gene STAD. (d) GO enrichment in GCSH-based STAD set analysis. (e) KEGG enrichment analysis in NFE2L2 gene STAD. (f) KEGG enrichment analysis in NLRP3-based STAD. (g) SLC31A1 gene STAD in KEGG enrichment analysis. (h) GCSH-based STAD in KEGG enrichment analysis.

The GO/KEGG annotation files and STAD tumor data files downloaded from the GSEA official website were read into R, and the enrichment analysis operation was performed to obtain the following: the GO of gene NFE2L2 in STAD is enriched in chromatin remodeling, DNA packaging, and protein DNA complex subunit organization function in Figure 9(a). The GO of gene NLRP3 in STAD is enriched in activation of immune response, adaptive immune response based on somatic recombination of immune receptors built from immunoglobulin superfamily domains, and alpha beta T cell activation functions in Figure 9(b). In addition, the GO of gene SLC31A1 in STAD is enriched in chromatin assembly or disassembly, epidermal cell differentiation, and inflammatory response to antigenic stimulus in Figure 9(c). And the gene GCSH is enriched in the GO of STAD in ribosome biogenesis, meiotic cell cycle, and intermediate filament cytoskeleton function in Figure 9(d). Finally, it was found that the gene NFE2L2 in the KEGG of STAD is enriched in olfactory transduction, circadian rhythm mammal, and graft versus host disease signaling pathway, as shown in Figure 9(e). The gene NLRP3 is enriched in the KEGG of STAD in the antigen processing and presentation, cytokine-cytokine receptor interaction, and cytosolic DNA sensing signaling pathway, as shown in Figure 9(f). In addition, gene SLC31A1 is enriched in olfactory transduction, cytosolic DNA sensing pathway, and regulation of autophagy signaling pathway in KEGG of STAD in Figure 9(g). The gene GCSH in the KEGG of STAD is enriched in olfactory transduction, arrhythmogenic right ventricular cardiomyopathy (ARVC), and hematopoietic cell lineage signaling pathway, as shown in Figure 9(h).

### 3.9. Potential Drug Screening

**Figure 10:**
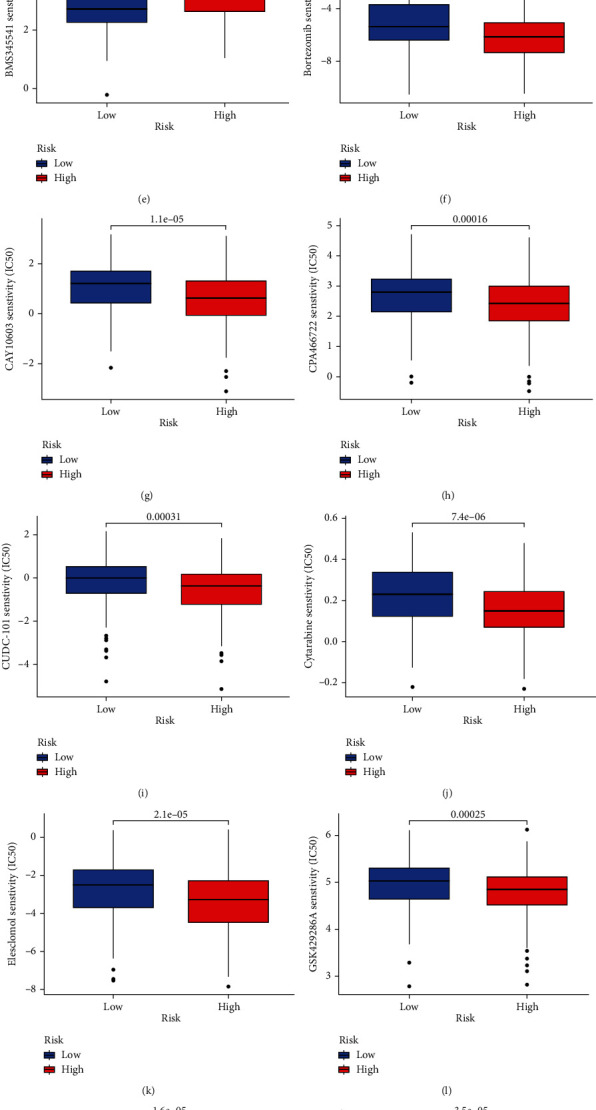
Potential drug screening. (a) AR-42 drug treatment effect. (b) Axitinib drug treatment effect. (c) Belinostat drug treatment effect. (d) BEZ235 drug treatment effect. (e) BMS345541 drug treatment effect. (f) Bortezomib drug treatment effect. (g) CAY10603 drug treatment effect. (h) CP466722 drug treatment effect. (i) CUDC-101 drug treatment effect. (j) Cytarabine drug treatment effect. (k) Elesclomol drug treatment effect. (l) GSK429286A drug treatment effect. (m) HG-6-64-1 drug treatment effect. (n) JW-7-24-1 drug treatment effect. (o) MG-132 drug treatment effect. (p) MLN4924 drug treatment effect.

According to the preliminary screening of GC cuprotosis-related risk genes, for the therapeutic effect of these genes, the CPG2016 drug database was used for screening, and the limma, ggpubr, pRRophetic, and ggplot2 packages were run to evaluate effective drugs for GC cuprotosis-related risk gene therapy. Discover AR-42, axitinib, belinostat, BEZ235, BMS345541, bortezomib, CAY10603, CP466722, CUDC-101, cytarabine, elesclomol, GSK429286A, HG-6-64-1, JW-7-24-1, MG-132, and MLN4924. Such drugs have good potential clinical efficacy on GC cuprotosis-related risk genes, as shown in Figures 10(a)–10(p).

## 4. Discussion

In this study, we obtained 407 transcriptome-related data sets and 443 clinically-related data sets through TCGA, explored the expression characteristics of 7 cuprotosis-related genes in GC, and constructed a prognostic model and its risk factors by constructing a prognostic model. Analysis, functional enrichment analysis, immune-related function analysis, and tumor mutational burden (TMB) analysis have confirmed for the first time that cuprotosis-related genes are related to the occurrence, development, and prognosis of GC.

Immunoregulatory factors and immune cells play important roles in the pathogenesis of GC [11, 12]. We first performed immune infiltration analysis of GC through the CIBERSORT package, an efficient analysis tool for gene expression consisting of 547 genes [13], which can characterize immune cell subtypes and accurately quantify different immune cell composition [14]. We found that the number of plasma cells, monocytes, and mast cells resting in GC tissue was significantly less than that in adjacent tissue, while the number of T cell CD4 memory activated macrophage M0, macrophage M1, and macrophage M2 which was significantly higher than that in adjacent tissue. It has been confirmed that GC is related to the regulation of immune infiltration, and previous studies have also shown that immune infiltrating cells are closely related to the occurrence and development of GC [15, 16].

R software was used to organize and summarize the clinical data set of GC obtained by TCGA and further obtain its cuprotosis-related expression matrix. According to *p* value < 0.05, the expression change range is ≥1.5 times (|log2 FC| ≥ 0.58), and a total of 7 cuprotosis-related differentially expressed genes (DBT, PDHB, CDKN2A, GLS, MTF1, NFE2L2, and DLST) were screened. Previous studies have found that PDHB is known to be closely related to colorectal cancer, renal cancer, and other malignant diseases [17, 18]. The expression of CDKN2A in small intestine adenocarcinoma is significantly different from that in colorectal cancer, but the expression of CDKN2A in GC is significantly different. Differential changes in expression have not been observed [19], while Tong et al. found that GLS is highly expressed in pancreatic ductal adenocarcinoma (PDAC) and demonstrated that SUCLA2-coupled regulated GLS succinylation can counteract oxidative stress in tumor cells [20]. In determining the targeted therapeutic potential of doxycycline in a cohort of GC patients, it was found that doxycycline activates MTF1-mediated transcription and inhibits histones, proteasome genes, fibroblast growth factors, and other oncogenic factors. The transcription of MTF1 and GC was further confirmed [21]. Previous studies have reported that NFE2L2 is highly expressed in GC and has anti-inflammatory and antioxidant effects [22–24], while DLST has been studied in neuroblastoma, breast cancer, and other tumor diseases [25, 26], and GC-related studies are rare.

We also randomly divided 443 clinical cases into a test group (Test) and a training group (Train) to construct a GC cuprotosis prognosis model and analyze its risk factors.

The results confirmed that both cuprotosis risk genes and tumor stage are important indicators for evaluating the prognosis of GC. At the same time, the related functional analysis of cuprotosis differential genes was carried out, and it was found that their pathways were mainly enriched in the TCA cycle and carbon metabolism in cancer. Gong et al. also found that the TCA cycle may be related to the pathogenesis of GC through GSEA enrichment analysis [27]. In addition, we also analyzed the immune-related functions of GC cuprotosis-related risk genes and confirmed that precise immunotherapy for GC cuprotosis-related risk genes can achieve the curative effect of GC. Tumor mutation burden (TMB) analysis of cuprotosis-related risk genes in GC showed that the high tumor mutation burden combined with low-risk group had the highest probability of survival, while the low tumor mutation burden combined with high-risk group had the lowest survival probability.

The core cuprotosis risk genes NFE2L2, NLRP3, SLC31A1, and GCSH were obtained through differential expression analysis and survival prognosis analysis in the early stage. We further studied the relative expression of core cuprotosis risk genes NFE2L2, NLRP3, SLC31A1, and GCSH in GC STAD and confirmed that NFE2L2 has low expression in STAD tumor tissue, while SLC31A1 and GCSH genes were highly expressed in STAD tumor tissue. There was no significant difference in the expression of NLRP3 between the two groups. At the same time, single-gene GSEA enrichment analysis was used to study the core cuprotosis risk genes in STAD tumor tissue. We performed gene enrichment analysis on STAD samples. SLC31A1 has been confirmed to be associated with various diseases such as lung cancer, ovarian cancer, and pancreatic cancer [28–30], but it has not been studied in GC. It has also been reported that GCSH is associated with the incidence of breast cancer, colorectal cancer, and papillary thyroid cancer [31–33]. No research has been conducted on the relationship between GCSH and GC. The research on NLRP3 and GC has been widely reported. Excessive inflammation driven by the NLRP3 inflammasome can promote GC progression [34]. Finally, we also obtained the risk genes related to cuprotosis in GC according to the previous screening and found AR-42, axitinib, belinostat, BEZ235, BMS345541, bortezomib, CAY10603, CP466722, CUDC-101, cytarabine, and elesclomol. Drugs such as GSK429286A, HG-6-64-1, JW-7-24-1, MG-132, and MLN4924 have good potential clinical effects on GC cuprotosis-related risk genes, which will help us to further study and explore treatment options for GC.

Our study still has limitations. First, although the expression of differentially expressed genes of cuprotosis in GC screened by TCGA proved that these genes are associated with the prognosis of the disease, a data set with a sufficiently large sample size and more complete clinical prognostic information is still needed for future research. Second, given that the predictions were established and validated by leveraging data from public databases, further biological evidence is required for validation in addition to the statistical evidence we provided. Finally, the specific mechanism of GC-related cuprotosis differential genes in the tumor immune process is still unclear, and further research is needed.

## Figures and Tables

**Table 1:** General clinical data of the two groups of patients.

Covariates	Type	Total	Test	Train	*p* value
Age	≤65	163 (43.94%)	84 (45.41%)	79 (42.47%)	0.7438
Age	>65	205 (55.26%)	101 (54.59%)	104 (55.91%)	
Age	Unknown	3 (0.81%)	0 (0%)	3 (1.61%)	
Gender	Female	133 (35.85%)	71 (38.38%)	62 (33.33%)	0.3655
Gender	Male	238 (64.15%)	114 (61.62%)	124 (66.67%)	
Grade	G1	10 (2.7%)	4 (2.16%)	6 (3.23%)	0.7102
Grade	G2	134 (36.12%)	65 (35.14%)	69(37.1%)	
Grade	G3	218 (58.76%)	112 (60.54%)	106 (56.99%)	
Grade	Unknown	9 (2.43%)	4 (2.16%)	5 (2.69%)	
Stage	Stage I	50 (13.48%)	24 (12.97%)	26 (13.98%)	0.7072
Stage	Stage II	111 (29.92%)	52 (28.11%)	59 (31.72%)	
Stage	Stage III	149 (40.16%)	80 (43.24%)	69 (37.1%)	
Stage	Stage IV	38 (10.24%)	20 (10.81%)	18 (9.68%)	
Stage	Unknown	23 (6.2%)	9 (4.86%)	14 (7.53%)	
T	T1	18 (4.85%)	7 (3.78%)	11 (5.91%)	0.7283
T	T2	78 (21.02%)	38 (20.54%)	40 (21.51%)	
T	T3	167 (45.01%)	83 (44.86%)	84 (45.16%)	
T	T4	100 (26.95%)	53 (28.65%)	47 (25.27%)	
T	Unknown	8 (2.16%)	4 (2.16%)	4 (2.15%)	
M	M0	328 (88.41%)	165 (89.19%)	163 (87.63%)	0.689
M	M1	25 (6.74%)	11 (5.95%)	14 (7.53%)	
M	Unknown	18 (4.85%)	9 (4.86%)	9 (4.84%)	
N	N0	108 (29.11%)	50 (27.03%)	58 (31.18%)	0.2444
N	N1	97 (26.15%)	45 (24.32%)	52 (27.96%)	
N	N2	74 (19.95%)	40 (21.62%)	34 (18.28%)	
N	N3	74 (19.95%)	44 (23.78%)	30 (16.13%)	
N	Unknown	18 (4.85%)	6 (3.24%)	12 (6.45%)	

## Data Availability

Data are available upon request from the authors. The data that support the findings of this study are available from the corresponding author (Kai Zhang), upon reasonable request.
